# Mathematical model for the tensile strength of the crimping assembly of aviation wiring harness end

**DOI:** 10.1038/s41598-021-97498-8

**Published:** 2021-09-09

**Authors:** Pin Li, Gongping Liu, Junhua Fan, Jiyuan Sun, Hang Xiu, Chunlin Tian

**Affiliations:** 1 School of Mechanical and Electrical Engineering, Changchun University of Science and Technology, Changchun, 130022 People’s Republic of China; 2 Changchun University of Science and Technology Chongqing Research Institute, Chongqing, 401135 People’s Republic of China; 3 AVIC Xi’an Aircraft Industry (Group) Company LTD, Xi’an, 710089 People’s Republic of China

**Keywords:** Engineering, Aerospace engineering, Mechanical engineering

## Abstract

In this study, the relationship between the tensile strength and the indentation depth was studied by analysing the deformation mechanism of the crimping assembly of the aviation wiring harness end. Tensile strength tests were performed on samples of crimping assemblies with different indentation depths. The results showed that the experimental and theoretical values were in good agreement, verifying the validity of the established mathematical model for tensile strength. Based on this model, a reasonable design range for the indentation depth corresponding to the specific combination of contacts and strands was determined.

## Introduction

**Figure 1:**
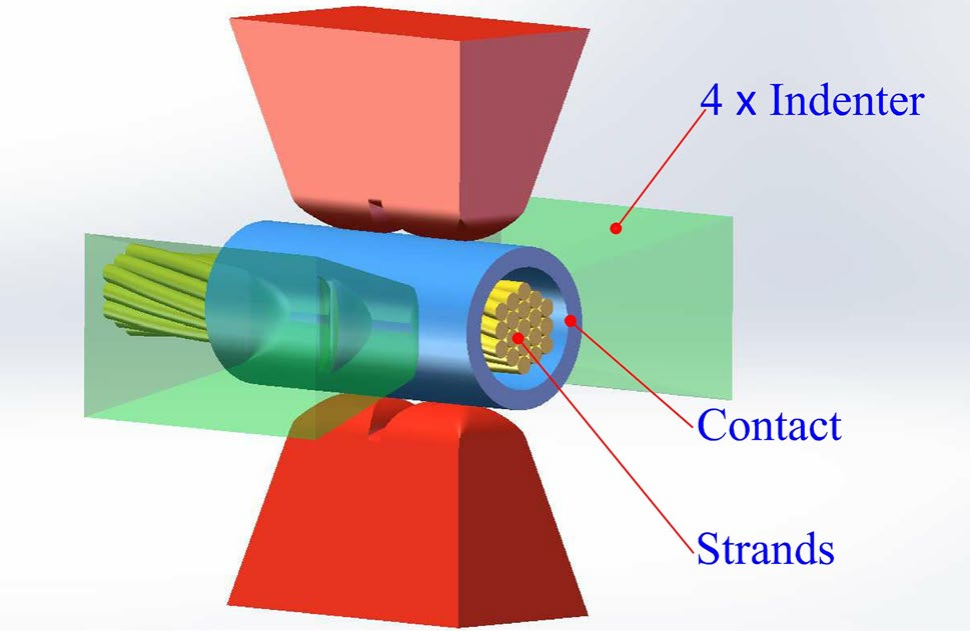
Four-indenter-eight-indentation crimping method for AWH end contacts.

Aviation Wiring Harnesses (AWH), which is the bridge for aircraft power supply and communication systems, is the foundation of any effective connection. In particular, the reliable processing quality of the AWH end guarantees the safe connection of the aircraft wire harness system. Numerous studies have focused on the processing technology of the AWH end, involving wire number recognition^1,2^, laser wire stripping^3,4^, and contact crimping^5–10^. In the traditional processing of the AWH end, contact crimping is the second production link after wire stripping. According to the MIL-DTL-22520G standard^11^, the crimping operation of the barrel contact studied herein should adopt the four-indenter-eight-indentation method, as shown in Fig. 1. The mechanical strength of the crimping assembly was closely related to the indentation depth. Although a large number of AWH crimping tools with different brands and diversified styles has emerged in the industry in recent years, most manufacturers only refer to a rough crimping specification for the design of indentation depth corresponding to different specific combinations of contacts and strands in the initial stage of product development^12^. To form the final product design specification in line with the industry standard, it is necessary to supplement a large number of tension tests of the crimping assembly in the later stage.

To avoid blindness in the design of contact indentation depth, extensive work has been devoted to the research of the crimping mechanism between the strands and the contact. Among them, the research method of three-dimensional numerical simulation analysis of the crimping process between strands and contact and the mechanical strength of crimping assembly is more popular. For example, Zhmurkin et al.^13^ conducted a three-dimensional nonlinear explicit dynamic simulation of the crimping process between 7-core strands and open-barrel contacts. The results showed that the elastic springback effect between the strands and the contact was the fundamental factor in forming a reliable crimp terminal after the tool pressure was released. However, the accuracy of the numerical model depends on the determination of the material behaviour law parameters. These parameters cannot be obtained through normal tensile or compression tests because the heat treatment and/or forming history of the crimping material can lead to the anisotropy of the material and change its mechanical properties, and the crimping materials have smaller dimensions. Mocellin and Petitprez et al. obtained the elastoplastic behaviour parameters with a linear power law of the crimping materials through the inverse analysis method using the FORGE software^14,15^, and established a numerical simulation model for the tensile strength considering the geometric shape and stress state of the material after crimping^16^; they also numerically studied the influence of parameters, such as material size and indentation depth on the tensile strength of the crimping assembly^17^. However, in the process of numerical research on the tensile strength of crimping assembly, different researchers have different pre- and post-processing setting methods in the finite element analysis process, resulting in certain differences in the results. This is not conducive to the formation of a unified and effective guiding reference for the design of the indentation depth. Therefore, in this study, the authors analysed the crimping mechanism of the crimping assembly, studied the relationship between the tensile strength of the crimping assembly and the indentation depth, and then determined the reasonable design range of the indentation depth corresponding to the specific combination of contacts and strands.

In this study, a mathematical model for the relationship between the tensile strength of the crimping assembly and indentation depth from two aspects (theoretical analysis and experimental comparison) is established. The morphological characteristics of crimped cross-sections with different indentation depths were observed to deduce the relationship between the tensile strength of the crimping assembly and the indentation depth from the perspective of geometry and mechanics. Tensile strength tests were conducted on samples of crimping assembly with different indentation depths to obtain the relationship between the actual tensile strength and indentation depth. The experimental value of the tensile strength that changes with the indentation depth is compared with the theoretical value to verify the accuracy and applicability of the established mathematical model.

## Materials and methods

### Materials and tools for crimping

**Figure 2:**
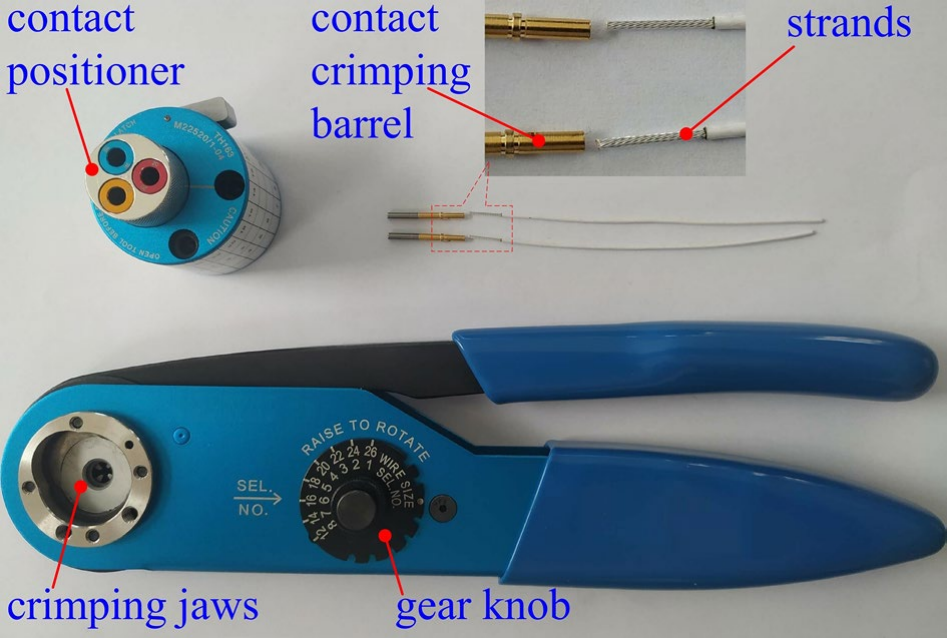
Crimping materials and tools.

Figure 2 shows the materials and tools used for crimping. The contacts were gold-plated copper materials with a size of 20# and a military product number of M39029/58-363, suitable for MIL-DTL-38999I II III series electrical connectors^18^, purchased from Huafeng Electrical Co., Ltd. (No. 188) (Guizhou, China). The size of the aviation wire was in accordance with the American wire gauge (AWG). The experiment used the size 22#, 19-core silver-plated copper strands that meet the MIL-DTL-22759 standards^19^ (purchased from Quanxin cable technology Co., Ltd. (Nanjing, China)). The crimping pliers (model YJQ-W2A) and contact positioner (model TH163) suitable for wire ranges of 12# to 26# meet the design requirements of the standard MIL-DTL-22520G^11^ for crimping tools. They were purchased from Jingrui Instrument & Equipment Co., Ltd. (Jiaxing, China).

### Test equipment for the crimping effect

The terminal pull-out force tester used to test the tensile strength of the crimping assembly was purchased from Xiangbang Automation Equipment Co., Ltd. (Shenzhen, China), with a maximum load of 500 N, an accuracy of 0.1 N, and a test range of 50 mm. The XY-M metallurgical microscope used to observe the deformation mechanism of the crimped cross-section was purchased from Sunny Optical Technology Co., Ltd. (Yuyao, China).

### Microscopic observation of crimped cross-section

By observing the crimped cross-section, to determine the deformation characteristics of the contact and the strands at the crimping position, the effect of analysing the crimping mechanism from both geometric and mechanical perspectives can be achieved. The experiment process is as follows: the 2, 3, and 4 position knobs of the crimping tool shown in Fig. 2 (indentation depth from large to small) were adjusted, and the positioner was rotated to the 20# contact position to make three crimp samples of the same material and different indentation depths. Subsequently, the cross-sections of the crimping parts of the three sets of crimping assemblies were obtained using the medium wire cutting equipment, and the cross-sections were polished. Finally, three sets of polished cross-sections of crimping were observed using a metallographic microscope, and the deformation morphologies of crimping assemblies with different indentation depths were compared.

### Testing experiment on tensile strength of the crimping assembly

**Figure 3:**
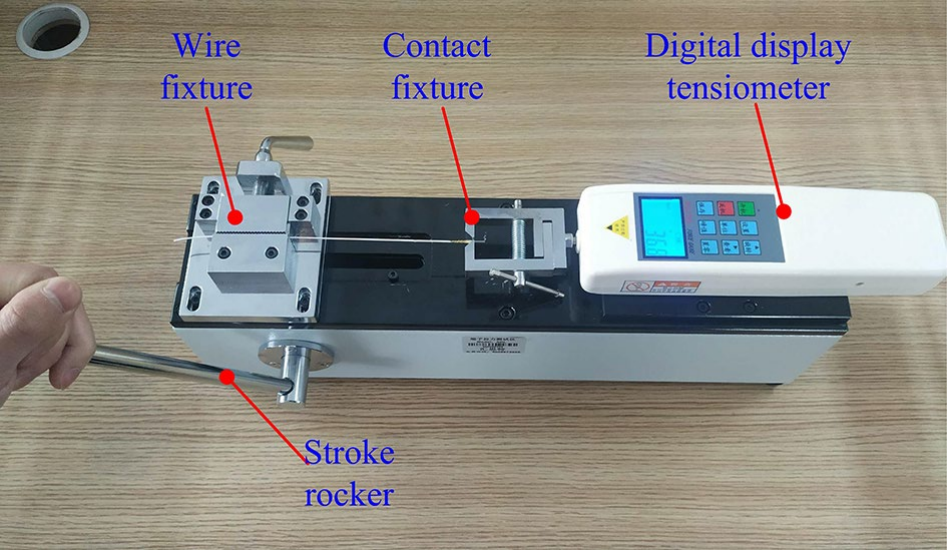
Test experiment on tensile strength of crimping assembly.

Through the tensile strength test of the crimping assembly samples, the actual value of the tensile strength corresponding to different indentation depths can be obtained. A comparison of the experimental and theoretical values can verify the accuracy of the mathematical model for tensile strength established in this study. The experiment process is as follows: gear knobs 1# to 5 # of the crimper were adjusted in turn, and five groups of five crimping samples were created in each group. The tensile strength test process of the crimping assembly is shown in Fig. 3. After the terminal pull-out force tester was assembled, the crimping sample contact was fixed to the right fixture of the equipment, the wire end was fixed to the left fixture of the equipment, and the sample was maintained at this level. The tension meter was set to the "maximum value" mode, the stroke rocker was rotated at a speed of about 50 mm/min, and the failure type of each sample and the maximum tension value after the sample was damaged was recorded.

### Analytical modelling of tensile strength of crimp assembly

When the indenter is released, both strands and contact have a large plastic deformation owing to mutual extrusion. The mechanical retention effect of the crimping assembly is mainly attributed to the residual contact force between the strands and the contact, which can be deduced inversely by analysing the interaction force between the wire cores in the strands based on the interaction principle of the forces.

### Geometric analysis of deformation mechanism of crimped cross section

**Figure 4:**
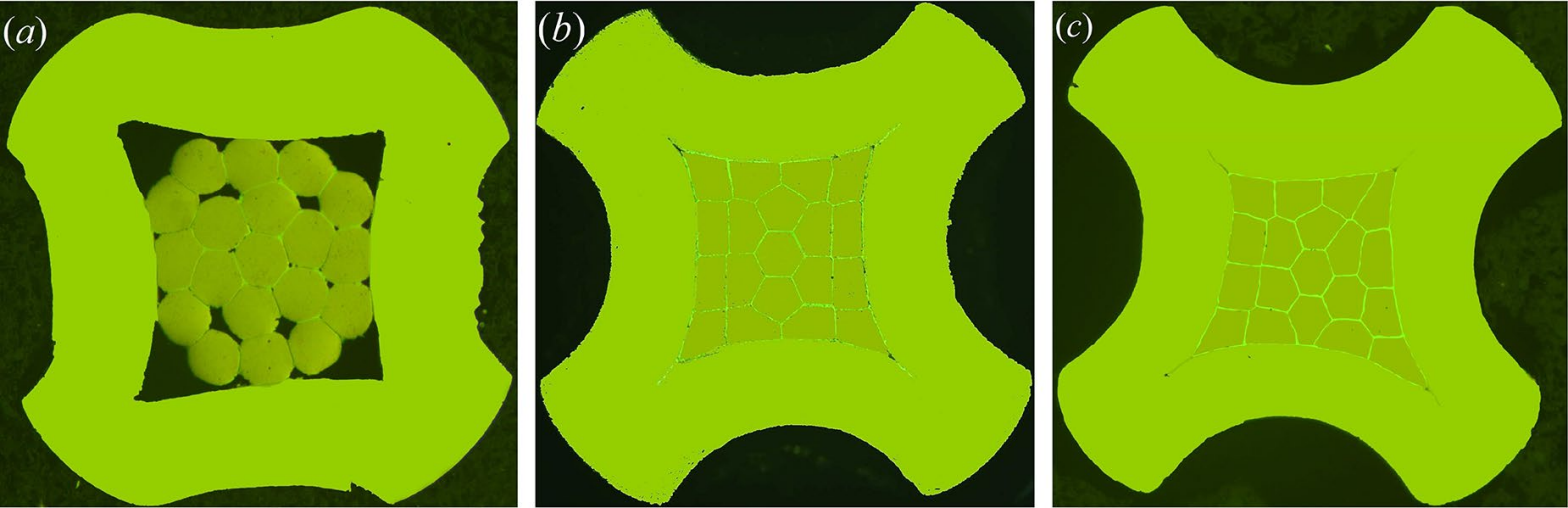
Morphological characteristics of crimped cross-sections with different indentation depths. (**a**) Insufficient crimping. (**b**) Reasonable crimping. (**c**) Excessive crimping.

Under the metallographic microscopic observation, the crimped cross-sections after polishing treatment showed three morphological characteristics, as shown in Fig. 4. The wire cores in Fig. 4a only have a smaller partial shape, which results in a large void ratio between the strands and the contact; thus, the tensile strength of the crimping assembly cannot meet the standard requirements. All the wire cores in Fig. 4b have a moderate deformation, and the strands and the inner surface of the contact piece crimp barrel are evenly squeezed with each other without gaps. The strands in Fig. 4c are excessively compressed, causing the wire cores to be in an excessively reduced diameter state. Under the load required by the standard, the strands are prone to unallowable breakage at the crimping place. Therefore, the crimp setting applied in Fig. 4b should be preferred.

**Figure 5:**
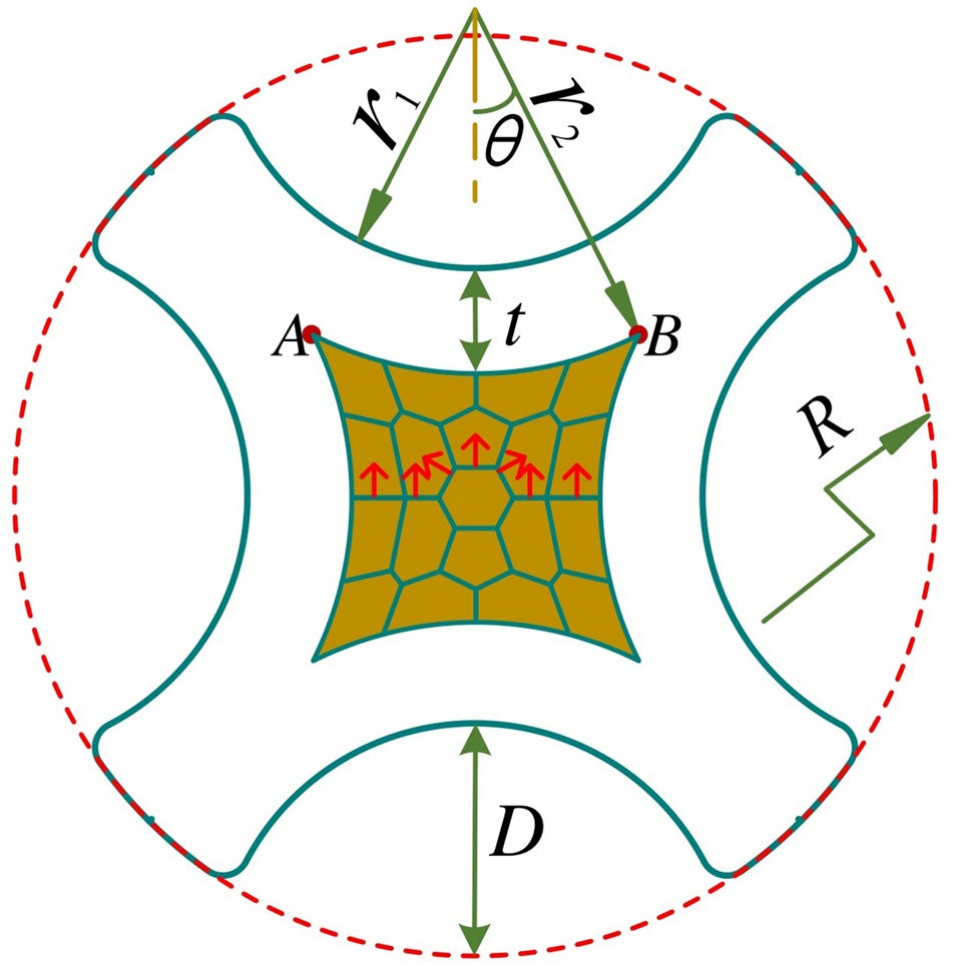
Schematic diagram of the 2*D* geometric dimension relationship of crimped cross-section.

To further analyse the geometric shape of the crimped cross-section, it is necessary to clarify the geometric dimension relationship between the strands and the contact after crimping. According to the morphological characteristics of the crimped cross-section shown in Fig. 4b, it is a reasonable analysis method to draw the 2*D* geometric dimension relationship graph of the crimped cross-section as shown in Fig. 5.

In the figure, points *A* and *B* represent the two adjacent meshing points on the inner surface of the contact crimping barrel after crimping, ${r}_{1}$ represents the radius of the cylindrical surface at the top of the indenter, ${r}_{2}$ is the radius of the arc *AB*, *t* is the wall thickness of the contact crimping barrel, *R* is the radius of the outer surface of the contact crimping barrel before crimping, *D* is the indentation depth, and *θ* is half of the central angle of the arc *AB*. In the actual crimping process, the contact wall thickness t changes slightly. To simplify the model, assuming that the size of *t* is ideally unchanged during the crimping process, and the inner cylindrical surface between the two points *A* and *B* is coaxial with the cylindrical surface at the top of the indenter, then $t={r}_{2}-{r}_{1}$, and the size of ${r}_{2}$ is a fixed value.

The total cross-sectional area *S* at the crimping position of the strands shown in the brown-gold area in Fig. 5 can be expressed by the following equation according to the geometric dimension relationship:

1 $$ \begin{aligned} & S = \left( {2r_{2} \cdot sin\theta } \right)^{2} - 4\left( {r_{2}^{2} \cdot \theta - r_{2} sin\theta \cdot r_{2} cos\theta } \right) \\ & = 4r_{2}^{2} \cdot \left( {sin^{2} \theta - \theta + \frac{1}{2}sin2\theta } \right) \\ \end{aligned} $$

After crimping, the relationship between the indentation depth *D* of the indenter and the corresponding *θ* can be expressed as follows:

2 $$ \begin{aligned} & D = R - t - \left[ {r_{2} \cdot sin\theta - \left( {r_{2} - r_{2} \cdot cos\theta } \right)} \right] \\ & = R - \left( {r_{2} - r_{1} } \right) + r_{2} - \sqrt 2 r_{2} \cdot sin\left( {\theta + \frac{\pi }{4}} \right) \\ & = R - \sqrt 2 \cdot r_{2} \cdot sin\left( {\theta + \frac{\pi }{4}} \right) + r_{1} \\ \end{aligned} $$

Thereafter, the angle *θ* can be expressed simultaneously as follows:

3 $$\theta =arcsin\left(\frac{R+{r}_{1}-D}{\sqrt{2}{r}_{2}}\right)-\frac{\pi }{4}$$

Assuming that the volume strain of each of the 19 wire cores in the strands after crimping is similar, the radial cross-sectional area of each wire core at the crimping position can be regarded as equal. The radial cross-section of the central wire core of the strands is approximately an ideal regular hexagon, and its area is represented by *W*. Subsequently, the total cross-sectional area *S* of the strands at the crimping position can be expressed as follows simultaneously:

4 $$ \begin{aligned} & S = 19W \\ & = 19 \cdot \left[ {6 \cdot \frac{{\left( {r_{3} - \delta } \right)^{2} }}{\sqrt 3 }} \right] \\ & = 38\sqrt 3 \left( {r_{3} - \delta } \right)^{2} \\ \end{aligned} $$

Here, ${r}_{3}$ is the radius of a single wire core before crimping, and *δ* represents the unilateral compression displacement of the central wire core of the strands after crimping.

By combining Eqs. (1) and (4), the unilateral compression displacement *δ* can be expressed as:

5 $$ \delta = r_{3} - r_{2} \cdot \sqrt {0.06\left( {sin^{2} \theta - \theta + sin\theta \cdot cos\theta } \right)} $$

### Elastoplastic mechanical analysis of the crimping assembly

Numerous studies have shown that the power hardening elastoplastic model is a widely used practical model for elastoplastic analysis of materials without obvious yielding platforms^20–23^. Mathieu petitprez et al. used the FORGE software to perform an inverse analysis of the mechanical properties of copper wire under radial compression^15^. The results show that the mechanical properties of copper wire exhibit a linear power-law relationship, and the stress-plastic strain relationship is as follows:

6 $$ {\upsigma } = \sqrt 3 K\left( {1 + a \cdot \varepsilon_{p}^{n} } \right) $$

Here, *σ* is the stress, ${\varepsilon }_{p}$ is the plastic strain, *K* is the rheological consistency coefficient of the material at yield, and *a* and *n* are the material hardening parameters. The values of *a, n*, and *K* are listed in Table 3.

Considering that the elastic strain ${\varepsilon }_{e}$ is much smaller than the plastic strain ${\varepsilon }_{p}$ in the process of radial compression and plastic deformation of copper wire, then the plastic strain ${\varepsilon }_{p}\approx\updelta /{r}_{3}$. Combining Eq. (5), Eq. (6) can be further expressed as:

7 $$ {\upsigma } = \sqrt 3 K \cdot \left[ {1 + a \cdot \left( {1 - \frac{{r_{2} \cdot f\left( \theta \right)}}{{r_{3} }}} \right)^{n} } \right] $$

Here $$f\left(\theta \right)=\sqrt{0.06({sin}^{2}\theta -\theta +\mathit{sin}\theta \cdot \mathit{cos}\theta )}$$.

**Figure 6:**
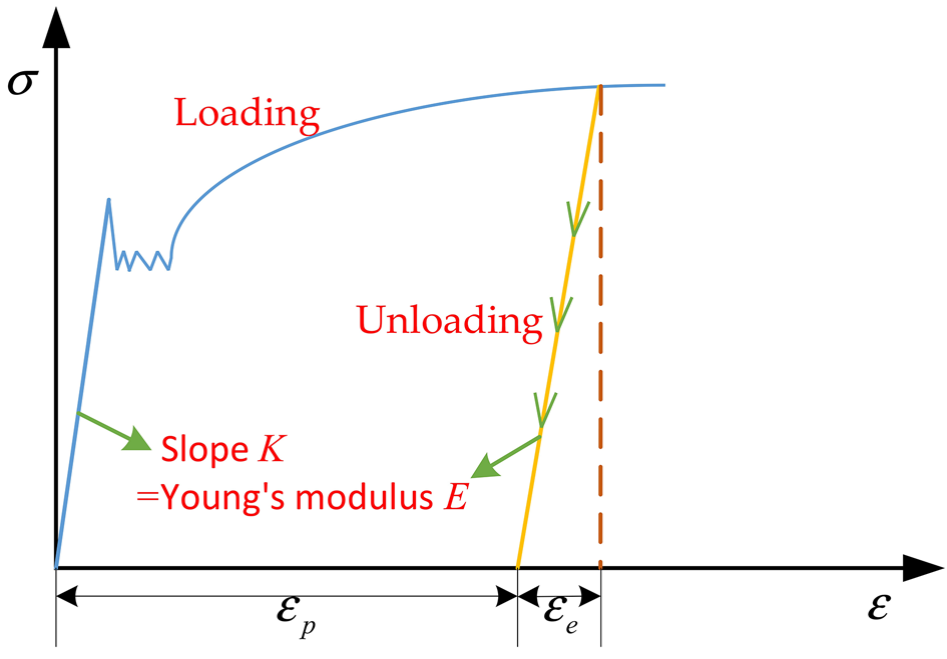
Stress–strain curves of loading and unloading for copper materials.

Research shows that after the load is released, the material undergoing elastoplastic deformation follows the law of elastic deformation during the original loading to reversely recover the part belonging to the elastic deformation^24–27^. The loading and unloading stress–strain curves of the copper material are shown in Fig. 6.

Here, ${\varepsilon }_{p}$ is the unrecoverable residual strain after unloading, and ${\varepsilon }_{e}$ is the strain recovered during the unloading process, $\varepsilon ={\varepsilon }_{p}+{\varepsilon }_{e}$. After the material enters the yield stage, if it is unloaded to zero from any point, the slope of the unloading line is the same as Young's modulus *E*.

According to the above analysis, the crimping force acting on the crimping assembly disappeared after the release of the indenter. Because there is a large gap between the contact crimping barrel and the strands before crimping, the contact had already undergone a large plastic deformation when it came into tight contact with the strands, and the hardness of the inner surface of the indentation was much higher than that of the surface of the strands. When performing unloading springback analysis on a crimped assembly, the contact can ideally be regarded as a rigid body. Each core in the strands has good plastic deformation and is in close contact with each other. It can be observed from the mutual extrusion arrangement characteristics of each wire core in Fig. 5 that, according to the force interaction and transmission principle, by analysing the unilateral springback displacement of the central wire core of the strands, the positive rebound force of the strands on the inner surface of the contact crimping barrel can be obtained indirectly, and the maximum static friction force between the strands and the inner surface of the contact can be reflected. The unilateral springback displacement *ω* of the central core can be expressed as follows:

8 $$ \omega = r_{3} \cdot \varepsilon_{e} = \frac{{r_{3} \cdot \sigma }}{E} $$

Combined with Eq. (7), Eq. (8) can be further expressed as:

9 $$ \omega = \frac{{\sqrt 3 \cdot r_{3} \cdot K}}{E} \cdot \left[ {1 + a \cdot \left( {1 - \frac{{r_{2} \cdot f\left( \theta \right)}}{{r_{3} }}} \right)^{n} } \right] $$

**Figure 7:**
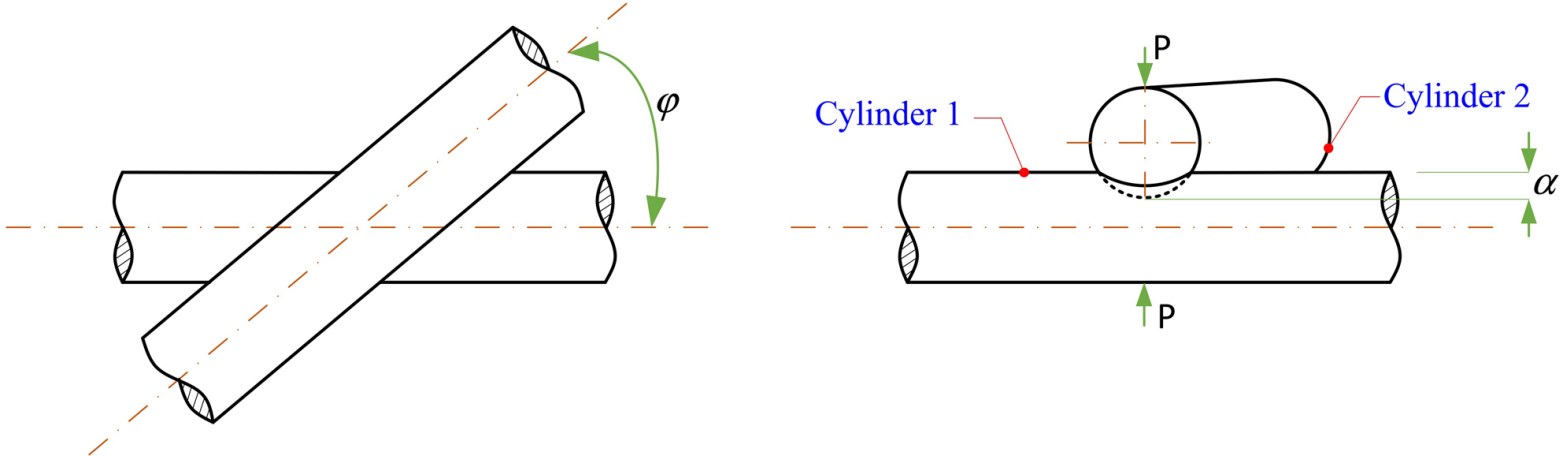
Radial elastic compression between equal-diameter cylinders whose shafts cross at any angle.

Because the cores in the strands are twisted with each other at a certain angle, when analysing the compression and springback characteristics between the wire cores, it is necessary to simplify the two adjacent wire cores into equal-diameter cylinders as shown in Fig. 7 where two shafts cross and radially squeeze each other at a certain angle. However, the general Hertzian elastic theory of line contact between two parallel cylinders is not applicable^28,29^.

In Fig. 7, *P* is the radial compression load, and *α* is the total radial elastic deformation of the two cylinders.

Reference^30^ provides the analytical relationship between *P* and *α* with respect to the elastic compression example shown in Fig. 7, and the analytical equation is as follows:

10 $$ \alpha = 2H \cdot \left[ {P \cdot \frac{{3\left( {1 - \nu^{2} } \right)}}{2\pi E}} \right]^{\frac{2}{3}} \cdot \left[ {\frac{A}{{2 \cdot \left( { - \frac{1}{e}\frac{dE*}{{de}}} \right)}}} \right]^{\frac{1}{3}} $$

Here, *ν* is Poisson's ratio; *E* is the Young's modulus of cylindrical radial compression, the value of *A* needs to be obtained by solving Eq. (11), and the ratio *A/B* is obtained; *e* is the eccentricity of the elliptical contact surface; *H* and $E*$ are the complete elliptic integrals of the first and second classes, respectively, with modulus *e*, and the values of *H* and $- \frac{1}{e}\frac{dE*}{{de}}$ can be further obtained from Table 3–6 of reference^30^ through the calculation value of *A/B*.

11 $$ \begin{aligned} & A + B = \frac{2}{D} \\ & \left( {A - B} \right)^{2} = \frac{{2\left( {1 + cos2\varphi } \right)}}{{D^{2} }} \\ \end{aligned} $$

Here, *D* is the diameter of the cylinder, and φ is the angle between the axes of the two cylinders.

According to the above analysis of the unilateral springback displacement *ω* of the central core in the strands combined with Fig. 7, it can be approximated as *α* = − 2*ω*. Combining Eqs. (9) and (10), the unilateral resilience force *F* generated by the central wire core after unloading can be expressed as follows:

12 $$ F = - P = \frac{2\pi E}{{3\left( {1 - \nu^{2} } \right)}} \cdot \left\{ {\frac{{\sqrt 3 \cdot r_{3} \cdot K}}{HE} \cdot \left[ {1 + a\left( {1 - \frac{{r_{2} \cdot f\left( \theta \right)}}{{r_{3} }}} \right)^{n} } \right]} \right\}^{\frac{3}{2}} \cdot \left[ {\frac{A}{{2\left( { - \frac{1}{e}\frac{dE*}{{de}}} \right)}}} \right]^{{ - \frac{1}{2}}} $$

Thereafter, according to the relationship between the angle *θ* and the indentation depth *D* described in Eq. (3), Eq. (12) can indirectly reflect the relationship between the wire core unilateral resilience force *F* and indentation depth *D*.

Assuming that the unilateral resilience force between any two adjacent cores in the strand is equal, according to the principle of force transmission, the positive springback contact force of the strands on the inner surface of any indentation of the contact is equal to the vector sum of the unilateral resilience forces of each core, as indicated by the red arrow in Fig. 5. Subsequently, through the given static friction coefficient *μ* between the strands and the contact, the maximum static friction force between the strands and the contact can be obtained, that is, the pull-off force of the crimping assembly *f* = $24F\cdot \mu$. Combined with Eq. (12), the final pull-off force *f* is expressed as follows:

13 $$ f = \frac{16\pi \cdot E \cdot \mu }{{\left( {1 - \nu^{2} } \right)}} \cdot \left\{ {\frac{{\sqrt 3 \cdot r_{3} \cdot K}}{HE} \cdot \left[ {1 + a\left( {1 - \frac{{r_{2} \cdot f\left( \theta \right)}}{{r_{3} }}} \right)^{n} } \right]} \right\}^{\frac{3}{2}} \cdot \left[ {\frac{A}{{2\left( { - \frac{1}{e}\frac{dE*}{{de}}} \right)}}} \right]^{{ - \frac{1}{2}}} $$

## Results and discussion

### Analysis of tensile test data of the crimping assembly

By adjusting the crimping gear of the crimping pliers, the minimum distance between the top ends of the opposing indenters shown in Fig. 1 is changed, thereby changing the indentation depth of the contact crimping barrel; the manufacturer design file of the YJQ-W2A crimping tool used provides a data description for this. Subsequently, according to the outer diameter of the 20# contact crimping barrel, the indentation depth range corresponds to the 1# to 5# crimping gear, as shown in Table 1.

**Table 1:** Indentation depth range corresponding to 1# to 5# crimping gear for 20# contact.

Gear (#)	Indentation depth (mm)
1	0.4709 ~ 0.5344
2	0.4201 ~ 0.4836
3	0.3693 ~ 0.4328
4	0.3312 ~ 0.3947
5	0.2550 ~ 0.3185

**Figure 8:**
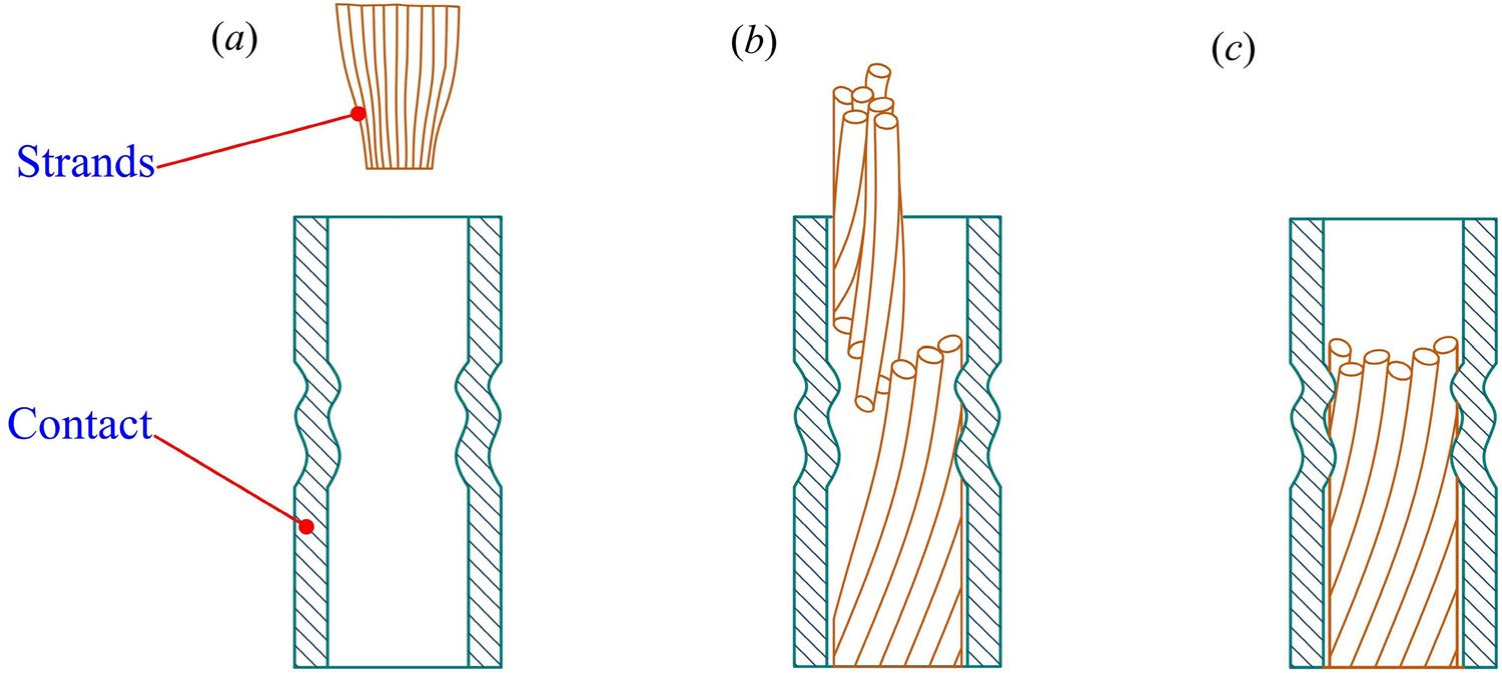
Types of failure in the tensile test of crimped samples. (**a**) Strands slide out of the contact crimping barrel. (**b**) Strands are partially broken at the crimping point, and some of the wire cores slide out of the contact crimping barrel. (**c**) Strands are completely broken at the crimping point.

According to the requirements of the MIL-DTL-22520G standard, the assembly must be able to withstand a tensile force of at least 60 N^11^. Table 2 lists the tensile test values of the first to fifth groups of crimping samples corresponding to the 1 # to 5*#* crimping gears and the corresponding crimping assembly failure types. The tensile strength of the fifth group of crimping samples did not meet the standard requirements. In the tensile test, the strands slide directly out of the contact crimping barrel, as shown in Fig. 8a. The tensile strength of the first to the fourth groups of crimping samples met the standard requirements. In the tensile test, the strands in the third and fourth groups of crimping samples were partially broken at the crimping point, and some of the wire cores slipped out of the contact crimping barrel, as shown in Fig. 8b. As the indentation depth increased, the crimped cross-section of the strands was further reduced. In the tensile test, as shown in Fig. 8c, the strands in the first and second groups of crimping samples were completely broken at the crimping point, and their tensile strength showed a downward trend compared with the third and fourth groups of crimping samples.

**Table 2:** Tensile test values of the first to fifth groups of crimping samples and the corresponding failure types of crimping assembly.

Gear (#)	Maximum tensile strength (N)	Average (N)	Type of destruction
1	73.2/76.4/74.5/73.8/76.1	74.8	Complete break
2	80.1/82.3/81.5/81.1/82.1	81.4	Complete break
3	83.3/83.7/83.6/82.8/83.5	83.4	Partial break
4	78.5/78.9/79.6/78.8/79.1	79.0	Partial break
5	24.7/25.3/24.7/25.8/26.1	25.3	Disengage

**Figure 9:**
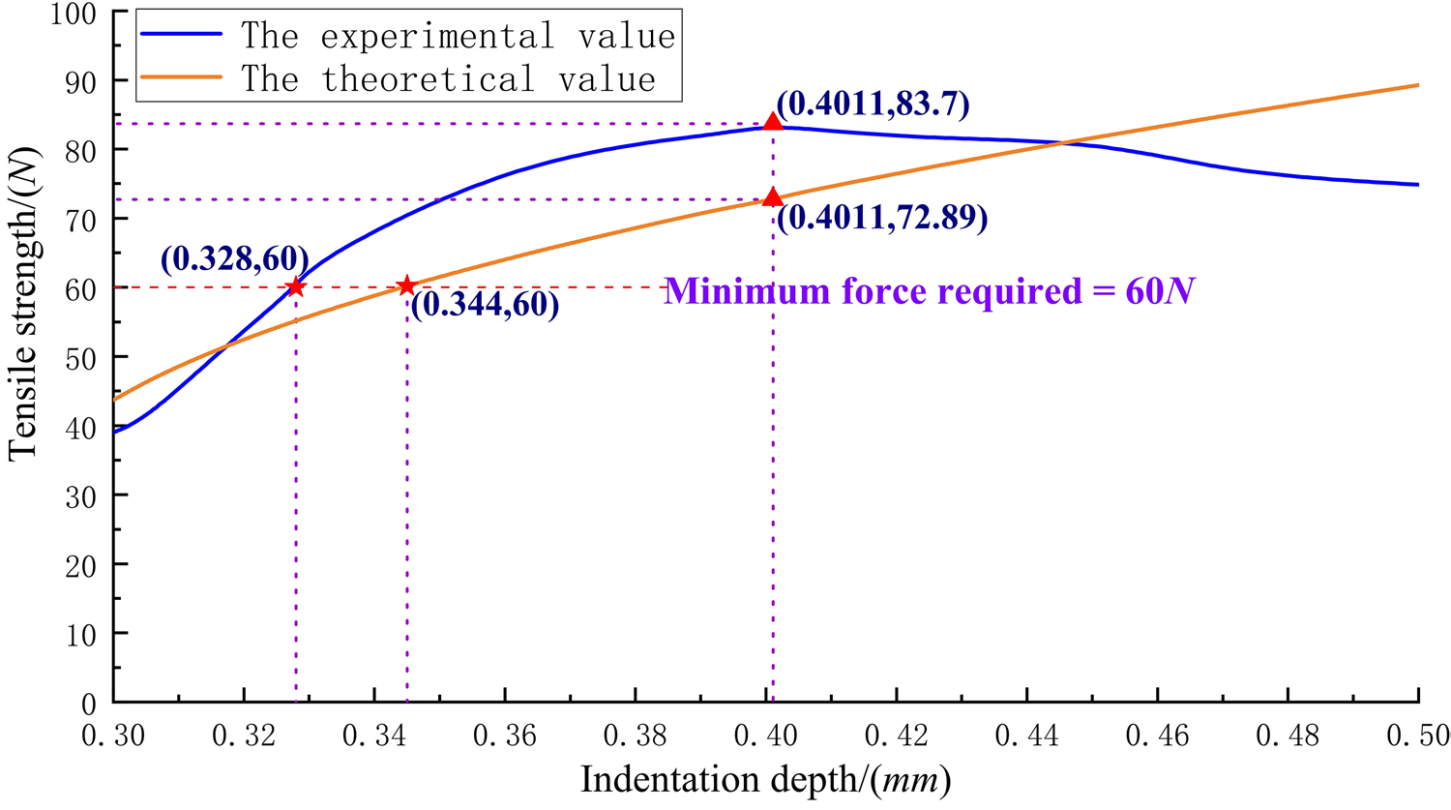
Tensile strength of crimping assembly that varies with indentation depth: the experimental and theoretical values.

Thereafter, the average tensile strength of five samples in each group was calculated by considering the change in the indentation depth as the abscissa, and the average value of the tensile strength at different indentation depths as the ordinate; the experimental curve of the relationship between the indentation depth and the tensile strength of the crimping assembly is drawn as shown in the blue curve in Fig. 9.

### Analysis of the modelling results of tensile strength of crimping assembly

The accuracy of the analytical model depends on the determination of the input data. Table 3 lists all the parameter data required by Eq. (13) to calculate the tensile strength of the crimping assembly. The relevant dimensions and mechanical parameters of the indenter, contact, and strands were provided by the material supplier. The unit of dimensions was in mm, Young's modulus of radial compression of the wire core was 50,000 MPa, and the value of the static friction coefficient *μ* was consistent with the typical friction coefficient value between two non-lubricating metals. The linear power-law elastoplastic mechanical behaviour parameters during the radial plastic deformation of the wire core can be obtained from the research results of reference^15^; the material yield rheological consistency coefficient *K* is assimilated into the threshold stress in MPa. Furthermore, the parameter *A* is obtained by Eq. (11) based on the diameter of the wire core and the twisting angle ϕ between the wire cores, and the values of *H* and $-\frac{1}{e}\frac{dE*}{de}$ can be further obtained by referring to the research data of the elastic contact mechanics of cylinders with intersecting axes in reference^30^.

**Table 3:** Related parameters of the analytical model for tensile strength of crimping assembly.

${r}_{1}$(mm)	*t* (mm)	${r}_{3}$(mm)	*R* (mm)	φ (°)	*ν*	*E* (MPa)
0.5	0.32	0.075	0.89	30	0.326	$5\times {10}^{4}$
*μ*	*K* (MPa)	*a*	*n*	*A*	*H*	$-\frac{1}{e}\frac{dE*}{de}$
0.37	98	1.28	0.46	0.893	3.1136	2.1408

Subsequently, by substituting the relevant data in Table 3 into Eq. (13), and calculating and solving it using MATLAB software, the theoretical curve of the relationship between the indentation depth and tensile strength, as shown in the orange curve in Fig. 9 is drawn.

### Determination of a reasonable indentation depth range

From the tensile strength experimental curve of the crimping assembly shown in Fig. 9, it can be understood that the indentation depth corresponding to the top of the curve is the optimal indentation depth of the set of crimping components. Considering the manufacturing errors of the contact, indenter, and strands, to avoid excessive crimping, the indentation depth suitable for the specific combination of the contact and the strands should be selected to the left of the indentation depth corresponding to the apex of the curve, and the tensile strength should not be less than 90% of the maximum value.

Excessive crimping causes serious shrinkage of the strands, and as a result, the strands produce a completely broken phenomenon, which is unallowable at the crimping place, even if it still has sufficient tensile strength. The mathematical model of tensile strength deduced in this study only considers the state in which the strands are pulled off or partially broken at the crimping position, and ignores the stage where the tensile strength decreases with the increase in indentation depth caused by excessive crimping. Therefore, the theoretical curve exhibits an increasing trend. Comparing the experimental and theoretical curves of the tensile strength of the crimping assembly shown in Fig. 9, it can be found that within a certain indentation depth range, the analytical model curve and the experimental curve exhibit the same trend. In addition, the relative error of the indentation depth of the two curves at the minimum tensile strength required by the standard was within 5%, and the relative error of the tensile strength of the two curves at the optimal indentation depth value shown in the experimental curve is less than 13%. Therefore, the accuracy and applicability of the established mathematical model for the tensile strength of a crimping assembly is acceptable.

In summary, without conducting the tensile strength test of the crimping assembly, for a specific combination of different contacts and strands, the design work of the new crimping tool on the indentation depth can refer to the mathematical model for the tensile strength of the crimping assembly derived in this study to obtain the theoretical value of the indentation depth corresponding to the minimum tensile strength required by each set of crimping components. The interval in which the value increases by 5% to 15% to the right can be regarded as a reasonable design range for the indentation depth.

## Conclusion

In this work, through the analysis of the deformation mechanism of the crimping assembly, the derivation of the relationship between the tensile strength and indentation depth was completed. Simultaneously, comparing the theoretical and experimental values of the tensile strength of the crimping assembly, it was found that they have the same force–displacement trend and smaller errors within a reasonable range of indentation depth, verifying the accuracy and applicability of the established mathematical model for tensile strength.

In summary, the mathematical model for the tensile strength of the crimping assembly derived in this study has a certain guiding significance for the product design of new crimping tools. However, when analysing the relationship between the tensile strength of the crimping assembly and the indentation depth, the influence of the difference in the shape of the indenter on the tensile strength value and the further requirements of the voltage drop of the crimping assembly on the indentation depth were not considered, and these factors need to be further studied in the near future.

## Data Availability

The datasets of related experiments involved in this article are available from the corresponding author upon reasonable request.
